# Screening, diagnosis and management of hypothyroidism in pregnancy: Number 10 – October 2022

**DOI:** 10.1055/s-0042-1758490

**Published:** 2022-11-29

**Authors:** Sara Toassa Gomes Solha, Rosiane Mattar, Patrícia de Fátima dos Santos Teixeira, Maria Izabel Chiamolera, Carlos Alberto Maganha, Alberto Carlos Moreno Zaconeta, Renato Teixeira Souza

**Affiliations:** 1 Policlínicas Municipal, Sorocaba, SP, Brazil; 2 Departamento de Obstetrícia, Escola Paulista de Medicina, São Paulo, SP, Brazil; 3 Universidade Federal do Rio de Janeiro, Rio de Janeiro, RJ, Brazil; 4 Escola Paulista de Medicina, Universidade Federal de São Paulo, São Paulo, SP, Brazil; 5 Faculdade de Ciências Médicas de São José dos Campos, São José dos Campos, SP, Brazil; 6 Universidade de Brasília, Brasília, DF, Brasil; 7 Universidade Estadual de Campinas, Campinas, SP, Brazil

## Key points

- Pregnancy places a metabolic overload on the maternal thyroid, especially in the first trimester, mainly because of the demand imposed by the conceptus. The fetal thyroid becomes functionally mature only around pregnancy week 20. Until then, the fetus depends on the transfer of maternal thyroid hormones (THs).
- Thyroid hormones are essential for the adequate fetal neurofunctional and cognitive development.
- Hypothyroidism brings higher risks of obstetric and fetal complications, namely, first-trimester miscarriage, preeclampsia and gestational hypertension, placental abruption, prematurity, low birth weight, and higher perinatal morbidity and mortality.
- Primary hypothyroidism (involvement of the gland with difficulty in producing and/or releasing TH) is the most common form of disease presentation, with the main etiology of Hashimoto’s thyroiditis of autoimmune origin.
- In about 85%-90% of cases of Hashimoto’s thyroiditis, antithyroid antibodies are present; the antithyroperoxidase (ATPO) is the most frequent.
- Positivity for ATPO is determined when circulating values exceed the upper limit of the laboratory reference. It implies greater risks of adverse maternal-fetal outcomes. Such a correlation occurs even in ranges of maternal euthyroidism.
- The critical point for the diagnosis of hypothyroidism during pregnancy is an elevation of thyroid-stimulating hormone (TSH). The measurement of free thyroxine (FT4) differentiates between subclinical and overt hypothyroidism. In subclinical hypothyroidism, FT4 is within the normal range, whereas in overt hypothyroidism, FT4 values are below the lower limit of the laboratory reference.
- Treatment of hypothyroidism is performed with levothyroxine (LT4) replacement with the aim of achieving adequate TSH levels for pregnancy.
- Some women have a previous diagnosis of hypothyroidism, and may or may not be compensated at the beginning of pregnancy. Even in compensated cases, the increase in LT4 dose is necessary as soon as possible.
- In the postpartum period, adjustment of the LT4 dose depends on the condition of previous disease, on the positivity for ATPO, and also on the value of LT4 in use at the end of pregnancy.

## Recommendations

- In places with full technical and financial conditions, TSH testing should be performed for all pregnant women (universal screening) as early as possible, ideally at the beginning of the first trimester or even in preconception planning. In places with less access to laboratory tests, screening is reserved for cases with greater risk factors for decompensation, namely: previous thyroidectomy or radioiodine therapy, type 1 diabetes mellitus or other autoimmune diseases, presence of goiter, previous history of hypo or hyperthyroidism or previous ATPO positivity. The TSH dosage should be repeated throughout pregnancy only in these cases.
- The diagnosis of hypothyroidism is made from the TSH value > 4.0 mIU/L.
- Pregnant women with previous hypothyroidism, overt hypothyroidism diagnosed during pregnancy or those with the above-mentioned higher risk factors for decompensation should be referred for risk antenatal care, preferably in conjunction with the endocrinologist.
- Overt hypothyroidism in pregnancy is identified when TSH > 10 mIU/L, and treatment with LT4 is readily recommended at an initial dose of 2 mcg/kg/day.
- TSH values > 4.0 mUI/L and ≤ 10.0 mUI/L require FT4 measurement with two diagnostic possibilities: overt hypothyroidism when FT4 levels are below the lower limit of the laboratory reference, or subclinical hypothyroidism when FT4 levels are normal. The treatment for subclinical hypothyroidism is LT4 at an initial dose of 1 mcg/kg/day, and the dose should be doubled upon diagnosis of overt hypothyroidism.
- In cases of TSH > 2.5 and ≤ 4.0 mIU/L, if there are complete conditions, ATPO should be measured. If positive (above the upper limit of normal), treatment with LT4 at a dose of 50 mcg/day is indicated. If conditions are not complete, the repetition of the TSH dosage should be done only for cases at higher risk. In these cases, treatment with LT4 will be established when TSH > 4.0 mIU/L at a dose of 1 mcg/kg/day; if needed, the dose can be adjusted after FT4 evaluation.
- Women with previous hypothyroidism should have their LT4 dose adjusted to achieve TSH < 2.5 mIU/L at preconception. As soon as they become pregnant, they need a 30% increase in LT4 as early as possible. In practice, they should double the usual dose on two days a week.
- Levothyroxine should be given 30-60 minutes before breakfast or three hours or more after the last meal. Concomitant intake with ferrous sulfate, calcium carbonate, aluminum hydroxide and sucralfate should be avoided.
- The target of LT4 therapy during pregnancy is to achieve a TSH value < 2.5 mIU/L. Once the therapy is started, monthly control must be performed until the mentioned goal is reached.
- In the postpartum period, women with previous disease should resume the preconception dose. Cases diagnosed during pregnancy in use of LT4 ≤ 50 mcg/day may have the medication suspended. The others should reduce the current dose by 25% to 50% and repeat the TSH measurement in six weeks. Cases of ATPO positivity are at higher risk of developing postpartum thyroiditis and de-escalation of LT4 should be performed as explained.

## Background

There is an increase in the stimulation of the hypothalamic-pituitary-thyroid axis during pregnancy through different mechanisms: 1

- Increase in serum concentration of estrogens accompanied by an increase in thyroid hormone-binding globulin (TBG) and consequent reduction in free fractions of THs;
- Greater iodine clearance;
- Greater degradation of THs by placental deiodinases;
- Increase in the serum concentration of human chorionic gonadotropin (hCG), which stimulates the thyroid tissue by cross-reacting with the TSH receptor that can generate goiter and gestational transient hyperthyroidism (GTT).

**Figure 1.**
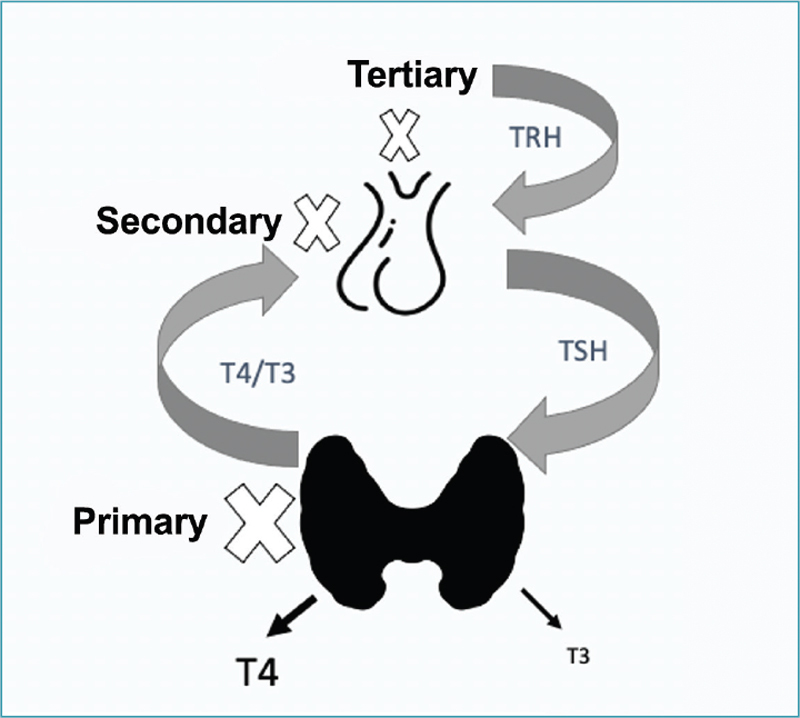
Schematic representation of the hypothalamic-pituitary-thyroid axis indicating the types of hypothyroidism. TRH: thyrotropin releasing hormone; TSH: thyroid stimulating hormone; T4: thyroxine; T3: triiodothyronine. **Source:** Prepared by the Working Group for Thyroid Dysfunctions in Pregnancy (Brazilian Society of Endocrinology and Metabology – SBEM).

This stimulus to the axis explains why pregnant women have lower TSH concentrations than non-pregnant women, especially in the first trimester. 1 In the first trimester of pregnancy, there are greater metabolic demands and given the changes in the stimulation of the gland, this a critical period for the occurrence of thyroid dysfunctions. All described changes in the physiology of the hypothalamic-pituitary-thyroid axis ensure the supply of THs to the fetus, especially in the period when the fetal thyroid is not yet functionally mature. Although the development of the gland begins at week 8, it functions fully only between weeks 18-20 of pregnancy. Therefore, until that moment, the fetus is totally dependent on the placental transfer of maternal THs. Note that this is a critical period for the formation of the fetal nervous system. 1 There is a compensatory mechanism, via feedback, between the thyroid, pituitary and hypothalamus that regulates glandular functioning. Knowing this mechanism helps in signaling where the cause of a possible dysfunction may be ( Figure 1 ).

Primary hypothyroidism is the most common form of the disease and occurs due to failure in the production and/or release of thyroxine (T4) and triiodothyronine (T3) hormones by the thyroid gland. 2 3 4 When central hypothyroidism is secondary to a failure in the production or release of TSH by the pituitary gland, manifestations from other pituitary sectors often occur. As gonadotropic or corticotrophic impairment is common, patients usually need to replace other hormones before they need TH replacement. 5 More rarely, central hypothyroidism has a tertiary origin resulting from hypothalamic disorders impairing the production or release of thyrotropin releasing hormone (TRH). In this document, we will address primary hypothyroidism during pregnancy. As in non-pregnant women, Hashimoto’s thyroiditis is its main cause, as well as the most prevalent among organ-specific autoimmune diseases, affecting about 12-13% of the population. 6 7 In approximately 85-90% of cases, Hashimoto’s thyroiditis presents with circulating antithyroid antibodies; the most common is the ATPO. 2 6 Primary hypothyroidism caused by Hashimoto’s thyroiditis may present with progressive thyroid failure, in which, initially, small elevations in serum TSH levels may compensate for slight reductions in the production of T4 and T3 by the thyroid. 3 4 Other causes of hypothyroidism include previous thyroidectomy and/or radioiodine therapy for definitive treatment of hyperthyroidism. Less commonly, primary hypothyroidism is caused by glandular infiltrative diseases (amyloidosis, hemochromatosis, sarcoidosis), drug use, and severe deficiency or excess of iodine. 2 3 4 A critical point in the investigation of hypothyroidism in pregnant women refers to what TSH elevation should be considered, defining the presence of primary hypothyroidism. When local reference values specific for pregnant women are unavailable, reference values from other centers that have been determined in a population of similar characteristics using the same TSH measurement methodology can be adopted. 8 According to the latest North American guideline, the upper limits of normality for TSH in the first trimester of pregnancy can be calculated by reducing 0.5 mIU/L from the upper limit of TSH reference values for non-pregnant women. 8 Such a strategy was confirmed in a study conducted with a population of pregnant women in the city of Rio de Janeiro. 9 Therefore, for most centers, the upper limit of TSH for pregnant women will be around 4.0 mIU/L, since most laboratory kits present a value of 4.5 mIU/L as the upper limit of normal for non-pregnant women. When treatment of primary hypothyroidism is indicated, LT4 replacement is required with the aim to reduce TSH levels to appropriate values for the patient’s age group and health condition. 2 3 4 These values are different for pregnant women compared to non-pregnant women in the same age group. 2 3 4 10

## Common scenarios in clinical practice

When addressing hypothyroidism in pregnancy, the main scenarios present patients previously diagnosed with hypothyroidism and those who received this diagnosis during pregnancy. The following conditions are possible for patients with a previous diagnosis:

- Under treatment (appropriate or not) with LT4;
- Untreated and decompensated;
- No treatment, but compensated, especially in cases of subclinical hypothyroidism.

In general, patients with hypothyroidism who get pregnant, even if compensated, need an increase in LT4 dose to maintain adequate TSH concentrations. Regarding diagnosis during pregnancy, we may have:

- Overt hypothyroidism;
- Subclinical hypothyroidism;
- Euthyroidism with positive ATPO.

The laboratory finding that combines TSH levels above reference values considered normal for a given population with normal circulating levels of THs is called subclinical hypothyroidism (mild or initial). 10 Overt hypothyroidism is considered when TSH levels during pregnancy are above 10.0 mIU/L 11 12 or when TSH levels are > 4 mIU/L and ≤ 10.0 mIU/L and the patient has TH levels below the lower limits of the respective reference values. 8 By knowing that most of the hormone produced by the thyroid is T4, which circulates preferentially linked to TBG and the latter is under direct interference from different factors, it is appropriate to measure FT4 as a way of estimating thyroid hormone production. Measurement of FT4 concentrations is performed to differentiate subclinical from clinical thyroid dysfunction. Obstetricians and endocrinologists must be familiar with thyroid function and dysfunction, as the correct diagnosis and treatment of thyroid diseases surely leads to better maternal and fetal outcomes.

## What are the possible maternal-fetal repercussions of hypothyroidism?

Overt hypothyroidism has been consistently associated with a higher risk of pregnancy complications, and impaired neurocognitive development of the conceptus. 8 12 13 Normal TH levels are essential for neuronal migration, myelination, and proper brain formation of the fetal brain. 8 14 15 Complications more commonly associated with overt hypothyroidism are: 8 11 13 16

- First trimester miscarriage;
- Preeclampsia and gestational hypertension;
- Placental abruption;
- Change in fetal vitality;
- Prematurity;
- Low birth weight;
- Cesarean delivery;
- Postpartum hemorrhage;
- Perinatal morbidity and mortality;
- Neuropsychological and cognitive impairment in children.

Although there are controversies regarding the causal relationship of subclinical hypothyroidism with all situations mentioned above, many studies indicate that women with subclinical hypothyroidism are also at a higher risk for:

- Preeclampsia;
- Premature birth;
- Placental abruption;
- Neonatal respiratory distress syndrome and/or pregnancy loss. 8 11 13

With regard to fetal neurocognitive development, overt hypothyroidism should be considered a risk factor for its alteration, and logic suggests that subclinical hypothyroidism, in the same way, can cause intellectual impairment in a variable spectrum. 8 However, some studies argue there is no neurocognitive benefit associated with treatment of subclinical hypothyroidism in pregnancy. 17 Some limitations of these studies, such as fixed or excessive dose of LT4, as well as late initiation of treatment should be considered. Further research is needed to determine if early initiation of treatment (before week 13) is beneficial. 11 17

Note that women with circulating ATPO, even with TSH within normal limits, are at a higher risk of pregnancy complications compared to women with negative antibodies. 8 12 13

Regarding the timing of delivery, there is no evidence to support the resolution of pregnancy before 40 weeks for women with hypothyroidism.

## In places with complete technical and financial conditions, for which pregnant women and when to request an evaluation of thyroid function?

Considering the possible complications of hypothyroidism during pregnancy, all women with pregnancy plans who known they have thyroid disease, should have their TSH levels evaluated, preferably before conception. On the other hand, there is no consensus on the best form of screening women without known thyroid disease. Despite the scarcity of robust evidence supporting universal screening, which proposes the evaluation of thyroid function for all pregnant women in the first trimester, several authors recommend this strategy under justification that different studies have shown the ineffectiveness of selective screening in diagnosing cases of hypothyroidism during pregnancy. 18 19 In a systematic review with meta-analysis, the two strategies were compared and almost half of cases of thyroid dysfunction went undiagnosed when only high-risk women were investigated. 20 In a recent study conducted in the city of Rio de Janeiro, 41.2% of pregnant women would remain undiagnosed because they did not meet any criteria for selective screening. 21 Thus, thyroid dysfunction during pregnancy presents high prevalence and is usually asymptomatic or oligosymptomatic. On the other hand, the serum TSH measurement, considered the test of choice for the diagnosis of thyroid dysfunction, is a sensitive, simple, robust, low-cost and widely available test, in addition to not offering risks to the patient when applied. Furthermore, the treatment of hypothyroidism, LT4, is an effective, inexpensive, available, and safe drug. 8 22 The benefits of treating overt hypothyroidism during pregnancy are crystal clear. According to recommendations of the World Health Organization (WHO), the only point that still lacks evidence for the complete inclusion of TSH in criteria establishing a screening test regards the lack of reported benefits of treating subclinical hypothyroidism during pregnancy. 22 Considering these premises, the present group recommends that under complete financial and technical conditions, the evaluation of thyroid function should be performed universally during pregnancy, as early as possible, at the beginning of antenatal care.

## In places where there are no complete financial and technical conditions, for which pregnant women and when to request the evaluation of thyroid function?

If technical and/or financial limitations exist, criteria should be used to select those at greater risk for developing hypothyroidism during pregnancy. Evaluation of thyroid function should be performed as early as possible, preferably at the beginning of the first trimester of pregnancy. According to the latest recommendation from the American Thyroid Association (ATA), the physician should identify patients at greater risk of developing thyroid dysfunction according to criteria presented in chart 1 . In these selected cases, TSH dosage will be recommended before conception or at the first antenatal visit. 8 Considering that some factors, especially age > 30 years and ≥ 2 pregnancies, significantly increase the number of pregnant women selected for screening, in situations of even more limited economic and technical conditions, this group suggests the priority evaluation of thyroid function for women with the following risk factors:

- History of head/neck irradiation;
- Prior thyroid surgery or radioiodine therapy;
- Type 1 diabetes mellitus or other autoimmune disease;
- Previously known thyroid autoimmunity or presence of goiter;
- History of previous hypo or hyperthyroidism.

**Chart 1.** Risk criteria for thyroid dysfunction during pregnancy according to the American Thyroid Association

*History of head/neck irradiation
*Previous thyroid surgery or radioiodine therapy
*Type 1 diabetes mellitus or other autoimmune disease
*Previously known thyroid autoimmunity or presence of goiter
*History of hypo/hyperthyroidism
- Signs/symptoms of thyroid dysfunction
- Morbid obesity (BMI ≥ 40 kg m²)
- History of fetal loss, premature birth or infertility
- Use of amiodarone or lithium, or recent administration of iodinated contrast
- Family history of autoimmune thyroid disease or thyroid dysfunction
- Resident in an area with moderate to severe iodine insufficiency
- Age > 30 years
- ≥2 pregnancies

BMI: body mass index

**Source:** Adapted from Alexander et al. 8

**Figure 2.**
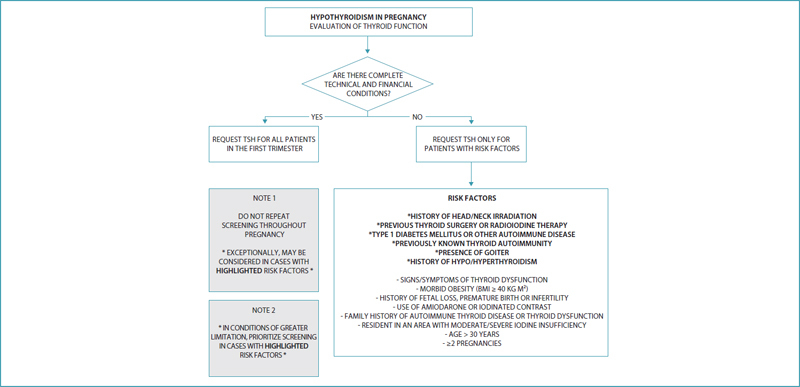
Hypothyroidism: evaluation of thyroid function. TSH: thyroid stimulating hormone; BMI: body mass index. **Source:** Prepared by the Working Group for Thyroid Dysfunctions in Pregnancy (CNEGAR and Brazilian Society of Endocrinology and Metabology – SBEM).

In the presence of symptoms such as fatigue, constipation, anemia and weight gain in addition to the expected during pregnancy, the possibility of evaluating thyroid function should be considered ( Figure 2 ).

## How to laboratory evaluate hypothyroidism?

Measurement of TSH is considered the test of choice for screening for thyroid dysfunction ( Chart 2 ). Given the known physiological reduction in TSH levels during pregnancy, elevations in these levels are highly sensitive and suggestive of dysfunction. Preferably, the trimester-specific reference values provided by the laboratory for the local pregnant population should be taken into account. However, this group suggests that in the absence of validated references in local populations, a TSH level ≤ 4.0 mIU/L should be considered as a normal value. When TSH levels are at concentrations above 10.0 mIU/L, the diagnosis of overt hypothyroidism is confirmed and the patient should be treated with LT4 immediately, regardless of FT4 levels. Thus, there is no need to measure FT4 or ATPO to guide management. If TSH values are > 4.0 mUI/L and ≤ 10.0 mUI/L, FT4 must be requested, with two possibilities:

- Subclinical hypothyroidism: when FT4 levels are normal;
- Overt hypothyroidism: when FT4 levels are below the lower limit of the laboratory reference.

**Chart 2.** Laboratory evaluation in the investigation of hypothyroidism in pregnancy

	TSH	T4L	Diagnosis	ATPO
HYPOTHYROIDISM DIAGNOSED PRIOR TO PREGNANCY	≤2.5 mUI/L	Do not request (Result will not change management)	Properly treated hypothyroidism during pregnancy	Do not request (Result will not change management)
	>2.5 mUI/L		Hypothyroidism not properly treated during pregnancy	
HYPOTHYROIDISM DIAGNOSED IN PREGNANCY	>10.0 mUI/L	Do not request (Result will not change management)	Overt hypothyroidism	Do not request (Result will not change management)
	>4.0 and ≤10.0 mUI/L	Within reference values informed by the laboratory	Subclinical hypothyroidism	Do not request (Proposed management in subclinical hypothyroidism is independent of ATPO)
	>4.0 and ≤10.0 mUI/L	Below the lower limit of reference informed by the laboratory	Overt hypothyroidism	Do not request (Result will not change management)
	>2.5 until ≤4.0 mUI/L	Within reference values informed by the laboratory	Euthyroidism	ATPO positive case (above the upper limit of reference informed by the laboratory), consider treatment
	≤2.5	Do not request (Result will not change management)	Euthyroidism	Do not request (Result will not change the proposed management for the first trimester in most patients)

TSH: thyroid stimulating hormone; FT4: free thyroxine; ATPO: antithyroperoxidase antibody; SCH: subclinical hypothyroidism.

**Source:** Prepared by the Working Group for Thyroid Dysfunctions in Pregnancy (CNEGAR and Brazilian Society of Endocrinology and Metabology – SBEM).

Therefore, the ideal is to have immediate access to FT4 levels to determine the management, as its value defines the subclinical or clinical condition of hypothyroidism. 8 12 13 However, if this access is unavailable, our group suggests immediate initiation of LT4 treatment.

- With TSH values > 4.0 mUI/L and ≤ 10.0 mUI/L and normal FT4, our group understands there is no need to request ATPO. Evidence regarding the benefits obtained with treatment of subclinical hypothyroidism when there is no circulating ATPO is weak, but these studies have methodological limitations and adequate LT4 replacement has low cost and few risks. Thus, we consider that treatment may be indicated in these patients.
- If TSH values are > 2.5 mIU/L and ≤ 4 mIU/L, ATPO should be tested. Justification is that when combined with positive ATPO (values above the upper limit of normality of the reference), this group indicates the beginning of treatment as early as possible, preferably at the beginning of the first trimester. These patients are at higher risk of TSH elevation. Studies point to the relevant role of autoimmunity in the occurrence of maternal-fetal adverse outcomes.

However, if ATPO testing is unavailable, the recommendation for the range of TSH > 2.5 mIU/L and ≤ 4 mIU/L, is as follows:

- It should not be treated;
- Patients who present the most relevant risk factors should have the TSH test repeated as soon as possible;
- If a TSH value > 4 mIU/L is identified, treatment with LT4 1 mcg/kg/day should be started and adjusted, if necessary, after FT4 evaluation.

## If the TSH level is normal in screening, should the test be repeated as the pregnancy progresses?

There is no need to repeat the evaluation of thyroid function throughout pregnancy. It should be performed at the beginning of antenatal care, preferably in the first trimester. If TSH concentrations are ≤ 2.5 mIU/L or > 2.5 and ≤ 4 mIU/L with negative ATPO, the patient is considered to be euthyroid. The dosage will only be repeated when there is any clinical suspicion of thyroid dysfunction or, exceptionally, in the presence of more relevant risk factors ( Figure 1 ).

## How to calculate the LT4 dose to start hypothyroidism treatment?

**Figure 3.**
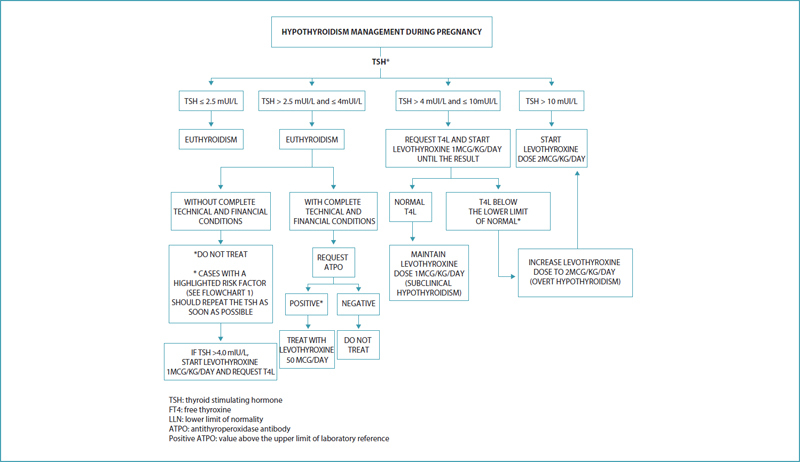
Hypothyroidism: management during pregnancy. **Source:** Prepared by the Working Group for Thyroid Dysfunctions in Pregnancy (CNEGAR and Brazilian Society of Endocrinology and Metabology – SBEM).

Recent studies have shown that the timing of introduction of TH can play an important role in the effectiveness of the intervention, and treatment in the first trimester has shown effectiveness in reducing the overall rate of pregnancy complications and the rate of preterm births. 23 24 25 Pregnant women with hypothyroidism should be treated with LT4 starting with doses of 1-2 mcg/kg daily. Since the objective is to reach euthyroidism as quickly as possible, the ideal is not to use the strategy of dose staggering, but to start with a full dose immediately. In a practical way, we recommend the following scheme ( Figure 3 ): 13

- TSH > 10 mUI/L: 2 mcg/kg/day;
- TSH > 4 mUI/L and ≤ 10 mUI/L with FT4 below the lower limit of the laboratory reference: 2 mcg/kg/day;
- TSH > 4 mUI/L and ≤ 10 mUI/L with T4L within the normal range: 1 mcg/kg/day;
- TSH > 2.5 and ≤ 4 mUI/L with positive ATPO: 50 mcg/day. 8 13

## What should be the type of antenatal care for pregnant woman with hypothyroidism?

Patients with hypothyroidism prior to pregnancy and those with a diagnosis of overt hypothyroidism in pregnancy screening should be followed up in high-risk antenatal care (preferably together with an endocrinologist). High surveillance is recommended for these patients. 8 Furthermore, in patients with more relevant risk factors for decompensation of thyroid metabolism during pregnancy, the same procedure should be followed. Such factors are:

- Prior head and neck irradiation;
- History of thyroid surgery and/or radioiodine therapy;
- Type 1 diabetes mellitus and other autoimmune diseases;
- Autoimmunity (positive ATPO) identified prior to pregnancy or presence of goiter;
- Previous history of hypo or hyperthyroidism.

In this differentiated follow-up, continuous interaction between obstetrician and endocrinologist is recommended, with serial laboratory measurements to obtain adequate and early metabolic control.

Cases of subclinical hypothyroidism without major risk factors should be followed up in Primary Care in usual risk antenatal care.

## How to perform dose adjustment for women with hypothyroidism who are planning to get pregnant or in early stages of pregnancy?

**Figure 4.**
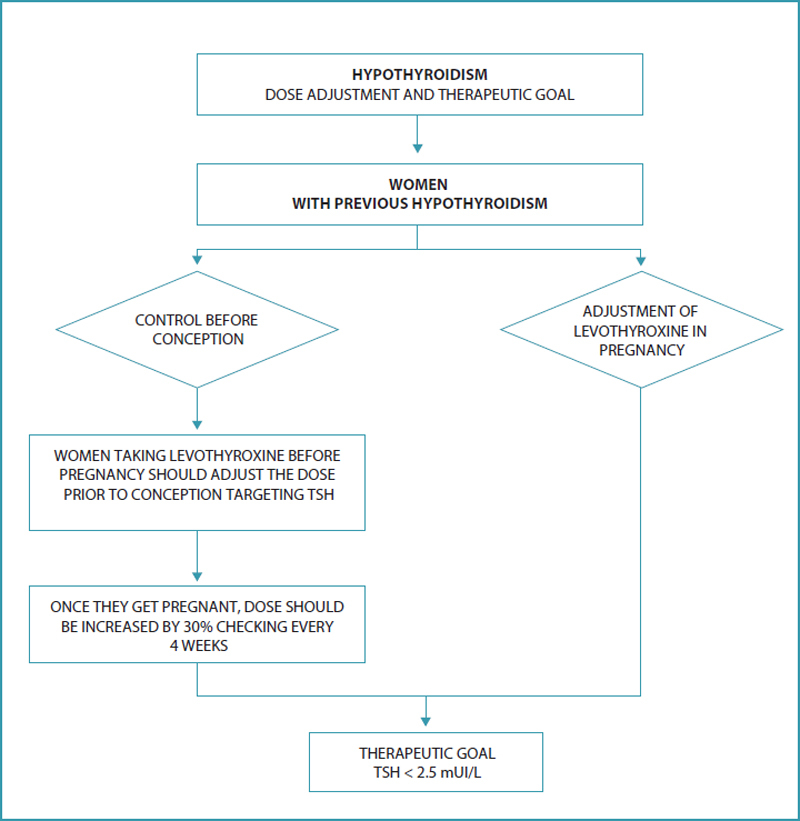
Hypothyroidism: dose adjustment and therapeutic goal. TSH: thyroid stimulating hormone. **Source:** Prepared by the Working Group for Thyroid Dysfunctions in Pregnancy (CNEGAR and Brazilian Society of Endocrinology and Metabology – SBEM).

Different cutoff points for TSH have been advocated as a target for preconception, ranging from < 1.2 mIU/L to < 2.5 mIU/L. It has already been found that only 17% of women with TSH < 1.2 mIU/L had to increase their LT4 dose during pregnancy. However, from a practical point of view, the recommendation so far is that women with hypothyroidism undergoing treatment optimize the dose in preconception, aiming to obtain TSH < 2.5 mIU/L ( Figure 4 ). 8 26 Pregnancy is associated with a higher need for TH in approximately one third of women already undergoing treatment. 26 27 This increased demand is believed to be related to greater estrogen production. 28 Clinical studies have confirmed that the higher need for exogenous LT4 occurs as early as weeks 4 to 6 of pregnancy. 26 27 This need gradually increases throughout weeks 16-20 of pregnancy and stabilizes thereafter until the time of delivery. For this reason, most pregnant women need to receive a higher dose of exogenous LT4 during pregnancy. 29 The importance of correcting doses early in pregnancy is noteworthy. The increase in LT4 dose varies depending on a number of factors:

- Underlying etiology of hypothyroidism: patients without functional thyroid tissue (following radiofrequency ablation or total thyroidectomy) have a greater need for dose increments compared to patients with autoimmune thyroid disease; 27
- The preconception TSH level: lower preconception TSH values can lead to lower TSH elevations during the first trimester; 26 30
- Variation in maternal estrogen levels during pregnancy correlates with variations in LT4 requirements during pregnancy; 27
- After pregnancy confirmation, LT4 adjustment should be done as soon as possible, without the need for a gradual increase. The recommendation is to increase the dose by about 30%. In practice, this is achieved by doubling the usual dose two days a week. The routine dose is maintained on the other days. This can effectively mimic gestational physiology and therefore, prevent maternal hypothyroidism during the first trimester.

## After starting treatment, what is the goal to be achieved? How often should exams be requested?

The treatment target is to achieve TSH in the lower half of the specific reference range of the pregnancy trimester. When this reference value is unavailable, it is reasonable to target maternal TSH concentrations below 2.5 mIU/L ( Figure 3 ). The TSH level should be evaluated every four weeks during dose adjustment. 8 13 31

## What should be the guidelines for prescribing LT4?

Preparations containing T3 should be avoided during pregnancy, as they increase T3 levels in comparison with T4, generating supraphysiological levels of maternal T3 and low levels of T4; maternal T4 is essential for the development of the fetal central nervous system. 8 As concomitant administration of LT4 and food can impair hormone absorption, LT4 administration is recommended 30-60 minutes before breakfast or at bedtime (three or more hours after the evening meal) for optimal and consistent absorption. In addition, LT4 should be taken separately from other potentially interfering drugs and supplements (eg ferrous sulfate, calcium carbonate, aluminum hydroxide, and sucralfate). 32 Switching LT4 brands can result in variations in the administered dose and should be avoided. 32 33

## How should postpartum dose adjustment be done?

**Figure 5.**
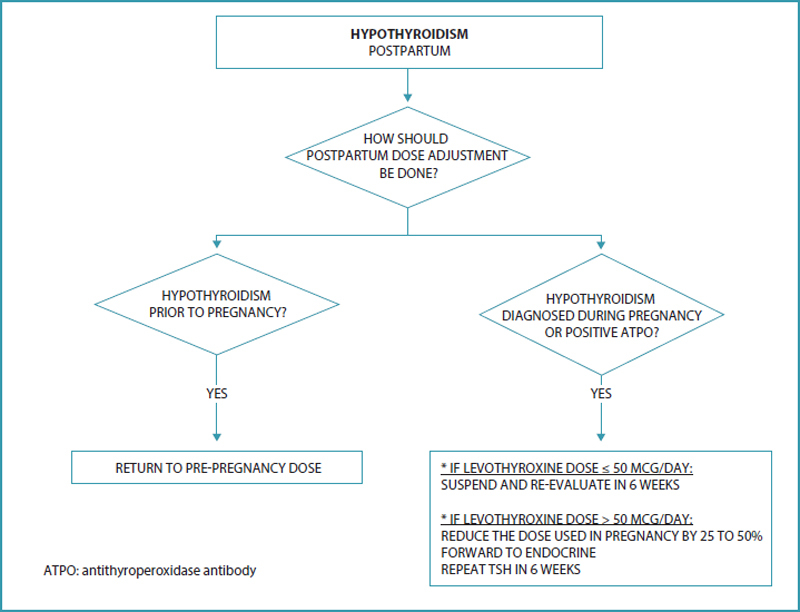
Hypothyroidism: postpartum. **Source:** Prepared by the Working Group for Thyroid Dysfunctions in Pregnancy (CNEGAR and Brazilian Society of Endocrinology and Metabology – SBEM).

After delivery, the maternal dose of LT4 should be adjusted to the dose used before pregnancy, and serum TSH should be measured after six weeks ( Figure 5 ). Some patients with Hashimoto’s thyroiditis who required an increase in the dose of TH during pregnancy may continue to need an additional dose in the postpartum period, in case of postpartum exacerbation of autoimmune thyroid dysfunction. On the other hand, some women with autoimmune thyroid disease who started taking LT4 during pregnancy may not need LT4 after delivery, especially when the dose is ≤ 50 mcg/d. If LT4 is discontinued, serum TSH should be re-evaluated in approximately six weeks. 34 When the dose used is greater than 50 mcg/d, a 25-50% reduction with further laboratory evaluation is recommended. 35

## Final considerations

Proper screening, diagnosis and management of hypothyroidism during pregnancy are essential, especially given the risks to fetal neurocognitive development and obstetric complications caused by improper treatment. Early identification is one of the major challenges, ideally at the beginning of the first trimester, allowing the initiation of therapy at the stage when the conceptus’ neuronal structures are developing. This way, the window of opportunity for real neurocognitive benefits for the offspring is reached. Thyroid hormones are essential for proper myelination and neuronal migration. The fetal thyroid is functionally mature only after week 20 of pregnancy; until then, the conceptus depends on the transfer of maternal THs. Furthermore, obstetric complications, especially those linked to first trimester abortion, preeclampsia, placental abruption and prematurity are at greater risk, especially when overt hypothyroidism is untreated. The laboratory test that guides the diagnosis of hypothyroidism is TSH, but there are several methodological limitations for the assertive establishment of TSH reference values representative of the local population. Current recommendations state that in this scenario, the normality value for TSH adopted during pregnancy should be ≤ 4.0 mIU/L. Universal screening is recommended provided that complete technical and financial conditions are available and should be carried out as early as possible. In adverse conditions, screening is reserved for cases with higher risk criteria for thyroid function decompensation during pregnancy. An important risk group is of women previously thyroidectomized or who underwent radioiodine therapy for the treatment of hyperthyroidism and who are already on LT4 therapy. Other critical factors include type 1 diabetes mellitus or other autoimmune diseases, the presence of goiter, a history of previous hypo or hyperthyroidism, or even the presence of autoimmunity. The T4L dosage helps to elucidate the overt or subclinical context of hypothyroidism. The role of autoimmunity –mainly ATPO positivity – is currently much studied. Women in this condition, even in euthyroidism, are at higher risk for adverse maternal-fetal outcomes. Although the literature is still controversial about the real benefits of treating subclinical hypothyroidism during pregnancy, evidence points to a relevant role of autoimmunity in the occurrence of potential complications in pregnancy and neurocognitive impairment of the offspring. Furthermore, the risk of occurrence of postpartum thyroiditis is greater in the presence of this autoimmune condition. In the preconception period, women who are known to be hypothyroid, especially those with positive ATPO, should be advised to adjust their LT4 therapy in advance, in order to enter the pregnancy-puerperal cycle under compensated conditions. De-escalation of LT4 in the postpartum period should be judicious and adequately monitored. It is a drug for use during breastfeeding. The challenges for coping with this pathology involve, above all, excessive diagnosis based on erroneous laboratory values that guide erroneous treatments and determine a non-existent gestational risk. At the other extreme, important conditions of thyroid dysfunction deserve adequate attention and guidance in high-risk antenatal care, receiving early and assertive treatments that avoid serious maternal-fetal complications.

National Commission Specialized in High Risk Pregnancy of the Brazilian Federation of Gynecology and Obstetrics Associations (Febrasgo)

President:

Rosiane Mattar

Vice-president:

Alberto Carlos Moreno Zaconeta

Secretary:

Mylene Martins Lavado

Members:

Arlley Cleverson Belo da Silva

Carlos Alberto Maganha

Elton Carlos Ferreira

Felipe Favorette Campanharo

Fernanda Santos Grossi

Inessa Beraldo de Andrade Bonomi

Janete Vettorazzi

Maria Rita de Figueiredo Lemos Bortolotto

Renato Teixeira Souza

Sara Toassa Gomes Solha

Vera Therezinha Medeiros Borges

Thyroid Department of the Brazilian Society of Endocrinology and Metabology

President:

Patrícia de Fátima dos Santos Teixeira (RJ)

Vice-president:

Danilo Glauco Pereira Villagelin Neto (SP)

Directors:

Rafael Selbach Scheffel (RS)

Cléo Otaviano Mesa Júnior (PR)

Gláucia Maria Ferreira da Silva Mazeto (SP)

Maria Izabel Chiamolera (SP)

Helton Estrela Ramos (BA)
